# Bronchopulmonary dysplasia from chest radiographs to magnetic resonance imaging and computed tomography: adding value

**DOI:** 10.1007/s00247-021-05250-1

**Published:** 2022-02-05

**Authors:** Nara S. Higano, Alister J. Bates, Chamindu C. Gunatilaka, Erik B. Hysinger, Paul J. Critser, Russel Hirsch, Jason C. Woods, Robert J. Fleck

**Affiliations:** 1 Center for Pulmonary Imaging Research, Cincinnati Children’s Hospital Medical Center, Cincinnati, OH USA; 2 Division of Pulmonary Medicine, Cincinnati Children’s Hospital Medical Center, Cincinnati, OH USA; 3 Department of Pediatrics, University of Cincinnati College of Medicine, Cincinnati, OH USA; 4 Division of Cardiology, Cincinnati Children’s Hospital Medical Center, Cincinnati, OH USA; 5 Department of Radiology, Cincinnati Children’s Hospital Medical Center, Cincinnati, OH USA; 6 Department of Radiology, University of Cincinnati College of Medicine, 3333 Burnet Ave., ML 5031, Cincinnati, OH 45229 USA

**Keywords:** Bronchopulmonary dysplasia, Chronic lung disease of prematurity, Computed tomography, Infants, Lungs, Magnetic resonance imaging, Preterm, Pulmonary hypertension, Radiography, Tracheobronchomalacia

## Abstract

Bronchopulmonary dysplasia (BPD) is a common long-term complication of preterm birth. The chest radiograph appearance and survivability have evolved since the first description of BPD in 1967 because of improved ventilation and clinical strategies and the introduction of surfactant in the early 1990s. Contemporary imaging care is evolving with the recognition that comorbidities of tracheobronchomalacia and pulmonary hypertension have a great influence on outcomes and can be noninvasively evaluated with CT and MRI techniques, which provide a detailed evaluation of the lungs, trachea and to a lesser degree the heart. However, echocardiography remains the primary modality to evaluate and screen for pulmonary hypertension. This review is intended to highlight the important findings that chest radiograph, CT and MRI can contribute to precision diagnosis, phenotyping and prognosis resulting in optimal management and therapeutics.

## Introduction

Throughout the world there are an estimated 15 million preterm births annually [1]. In the United States, 450,000 babies are born prematurely each year [2], with a 10–12% rate of premature birth. For comparison, most other modern, affluent countries have rates in the 5–8% range. Reduction of preterm birth is a national public health priority. Yet, after years of decline, preterm birth rate has climbed for the last 5 years [2].

Imaging has played a role in the care of preterm births for decades in the form of radiographs, US, fluoroscopy, CT, MRI and to a lesser extent nuclear medicine and angiography. Preterm birth affects all organs, although most of the morbidity and mortality is related to the respiratory system: lungs, airways and pulmonary vascular components. Infants born too early develop infantile respiratory distress syndrome, which is also called surfactant deficiency disorder, and in older literature hyaline membrane disease. Clinical care prioritizes keeping the infants alive so they can continue to grow and mature their organs, but the necessary respiratory support strategies expose the lungs to oxygen, mechanical ventilation pressures, excessive inflation and atelectasis. These traumas and lung development outside the uterus result in aberrant respiratory system development and growth arrest, causing chronic changes called bronchopulmonary dysplasia (BPD) or chronic lung disease of prematurity [3].

Since BPD was first described in 1967, respiratory care has evolved, modes of respiratory support have improved and surfactant therapy has been introduced [4]. This has resulted in improved survival in ever younger and smaller preterm babies. As a result, the rate of preterm births developing BPD still affects a substantial portion of preterm infants who require respiratory support or have respiratory morbidity after discharge [5].

Portable chest radiograph and chest CT in the evaluation of these children has always been used as a problem-solving tool. However, recent studies have begun to show that CT and MRI of the respiratory system can provide information that is not evident on chest radiograph, thereby providing diagnostic and prognostic information that influences care [6]. Additionally, over the last 20 years, efforts to reduce radiation dose of CT have improved the risk-to-benefit equation [7] and MRI is now becoming a viable method of evaluating the lungs because of ultrashort echo-time (UTE) and other three-dimensional (3-D) high-resolution, proton-density-weighted methods [8, 9]. In this review, we discuss the literature and present information that radiologists can use in evaluating the respiratory system in preterm infants in the post-surfactant era with chest radiograph, and the added value of CT and MRI.

## Bronchopulmonary dysplasia, defined

**Fig. 1:**
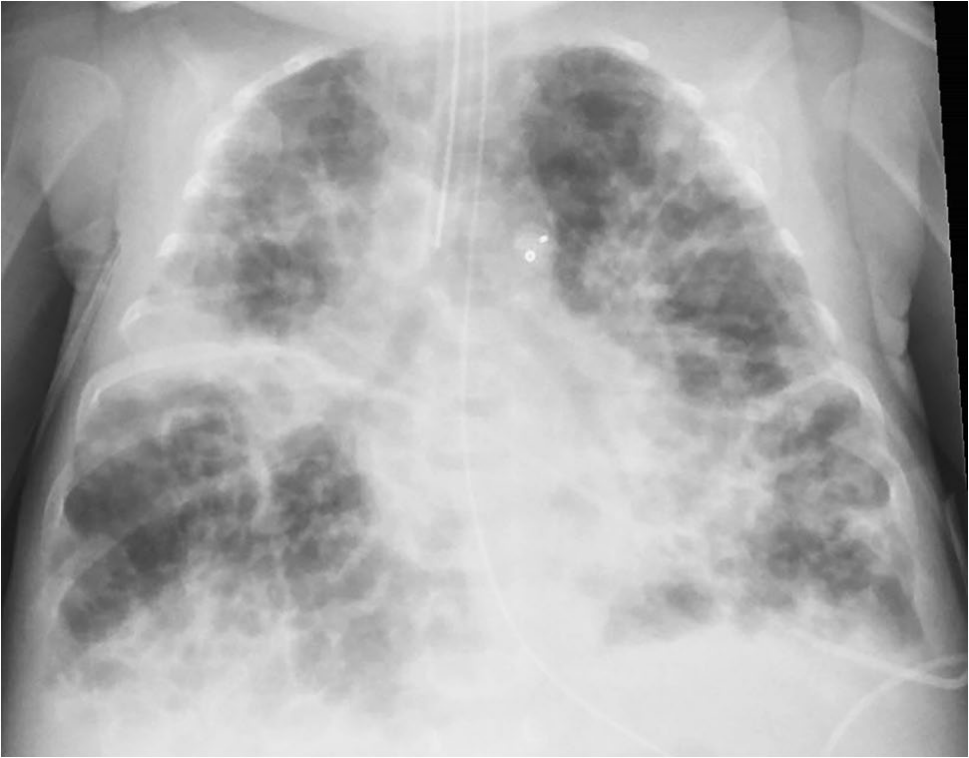
Typical appearance of bronchopulmonary dysplasia (BPD). Anteroposterior (AP) chest radiograph of a girl born at 25 weeks of gestation who is now post-menstrual age 36 weeks (11 weeks old) with severe BPD. The girl is still intubated and has a ductus arteriosus closure device. The lungs are characterized by overall hyperinflation, with mixed areas of density and hyperlucency characteristic of the AP chest radiograph appearance of severe BPD

A persistent challenge in the study and treatment of BPD is the lack of an effective definition, in part because of the multifactorial mechanisms of this unique disease. BPD was first described by Northway et al. [10] based on radiographic (Fig. 1) and pathological evidence of pulmonary disease (opacification, atelectasis, cysts, lucencies). While varying definitions have been proposed since then, they remain mostly operational, relying on degree and duration of oxygen requirement rather than pathophysiological condition [11].

In 1988, Shennan et al. [12] followed 600 preterm infants with birth weights <1,500 g for 2 years and determined that an oxygen requirement at 36 weeks post-menstrual age (PMA) was predictive of abnormal pulmonary function in the first 2 years after birth. However, there was increasing recognition through the 1990s that improvements in antenatal and postnatal treatment strategies were changing the pathophysiology of BPD (so-called new BPD) — namely, very-low-birth-weight infants with seemingly mild lung disease in early neonatal life were developing increasing ventilatory needs and undergoing pronounced alveolar and vascular “growth arrest” [13]. In response, a new diagnostic definition was proposed at a workshop in 2000 sponsored by the National Institutes of Health (NIH) National Heart, Lung and Blood Institute (NHLBI) and published in 2001 [13]. This 2001 consensus definition included graded severity levels based on gestational age and level of required respiratory support. Specifically, infants diagnosed with BPD require oxygen therapy for at least 28 cumulative days, with mild, moderate and severe grades determined by degree of respiratory support required at 36 weeks of post-menstrual age; while initially helpful in delineating severity, many infants eventually became unclassifiable with updated clinical practices such as high-flow nasal cannula with 21% oxygen or low-flow with 100% oxygen. Although additional clinical definitions have attempted to improve upon this definition (e.g., a physiological challenge of supplemental oxygen withdrawal) [14–16], the 2001 consensus definition has been implemented most widely, despite the inconsistent relationship between various BPD definitions and clinical outcomes [17–19].

A new definition was proposed in 2018 at a conference conducted by the NIH Eunice Kennedy Shriver National Institute of Child Health and Human Development (NICHD) to incorporate newer modes of noninvasive ventilation and address several deficiencies, including institutional variability, lack of evidence-based guidelines, and unclassified status of prematurity-related deaths prior to 36 weeks of post-menstrual age [3]. This 2018 definition asserts that a premature infants (<32 weeks of gestational age) with BPD have persistent parenchymal disease and use respiratory support at 36 weeks of post-menstrual age (grades I, II or III based on fraction of inspired oxygen [FiO2] range/oxygen level/oxygen concentration). Importantly, this refined BPD grading system reincorporated radiographic confirmation of parenchymal lung disease. Like previous definitions, this 2018 workshop definition struggles with early identification of infants who will eventually have severe chronic lung disease of prematurity; imaging performed early in the neonatal intensive care unit (NICU) might allow clinicians to apply targeted therapies to the most at-risk infants.

## Chest radiograph

Chest radiograph has always been at the forefront of decision-making in infants with respiratory distress. Northway et al. [10] first reported four stages of chest radiograph findings that can occur in preterm infants and coined the term BPD. By today’s standards the preterm infants were relatively large and at relatively advanced gestational ages. Since that time, treatment of mothers at risk of preterm birth and infants born preterm has evolved greatly and continues to evolve, as does the appearance of the chest radiograph.

The use of chest radiographs in infants for problem-solving is well established. However, over the years chest radiography has been proposed as part of the BPD definition and used to predict clinical outcomes such as growth, respiratory distress and gas exchange [20, 21]. In 1984, Toce et al. [21] developed a scoring system that included lung expansion, interstitial densities, focal emphysema (cysts) and cardiovascular abnormalities. Interestingly, they suggested that 2 weeks after birth is the most specific time to diagnose BPD [21]. Several authors in the 1980s noted that the evolution of chest radiography was nonlinear and appeared to be resulting from the changing treatments, including use of antenatal steroids in mothers, changing ventilation strategies and postnatal steroid treatments.

**Fig. 2:**
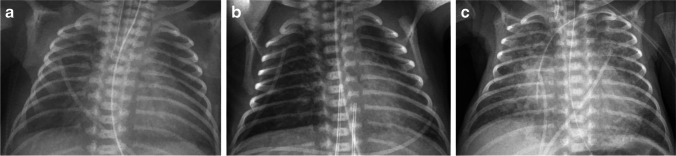
Evolving lung appearance on radiography in a premature newborn. **a** Anteroposterior (AP) chest radiograph in a 1-day-old boy born at 24 weeks of gestation shows diffuse, fine granular opacities throughout the lungs, often with air bronchograms. **b** AP chest radiograph of the same boy at 2 days old. Surfactant had been administered and the boy was extubated. At this time the lungs appear clear and better inflated. **c** AP chest radiograph in the same boy at 4 days of age shows that his lungs have become much more opaque and coarse, with mixed lucent and opaque areas resulting in reintubation

However, the most major change was noted when exogenous surfactant became clinically available in the early 1990s [22, 23], as described by Swischuk et al. [24]. The first radiograph demonstrated the granular appearance typically associated with preterm birth and surfactant deficiency, and 76% of infants cleared the granular opacities by 1 day old (Fig. 2). Subsequently, 45% remained clear and did well, but these also tended to be the larger infants with a more mature gestational age. The remaining 55% of infants developed hazy to opaque density in the lungs (Fig. 2) by the second week after birth, termed “leaky lung syndrome.” Only 11% of these became clear again. Lungs that did not clear went on to develop the cystic bubbly lucencies that are now typically associated with BPD (Fig. 1) [24].

Chest radiography studies looking at future growth noted that hyperinflation worsened along with focal areas of emphysema and interstitial opacities at 1 year compared to 1 month [25–27]. Moya et al. [28] investigated the ability of the chest radiograph obtained at 36 weeks of post-menstrual age to predict BPD by using a grading system and found that low scores were less reliable in predicting BPD outcome, but prediction improved with increasing scores. Kim et al. [29] found that an interstitial or coarse interstitial pattern (Fig. 2) at day 7 after birth was predictive of BPD developing with high specificity but low sensitivity, but that birth weight, gestational age and invasive ventilation were much more sensitive. More recent studies have related the cystic bubbly changes to a higher need for oxygen therapy [30], and a large study by Arai et al. [31] with a cohort of more than 8,000 infants showed that bubbly/cystic changes at 28 days was more predictive of home oxygen therapy than gestational age, birth weight, chorioamnionitis or any other factor. Those without the bubbly/cystic changes had only 2% rate of home oxygen. Bubbly/cystic change is also associated with wheezing [32, 33].

In sum, the chest radiograph of preterm infants starts with a granular pattern on day 1, often clears with administration of surfactant and then a substantial number of preterm infants develop diffuse opacity in the second week. As that diffuse opacity evolves into an interstitial, coarse or bubbly pattern, it becomes more predictive of BPD and outcomes after hospitalization. Although the coarse, interstitial bubbly pattern is specific, it lacks sensitivity.

## Computed tomography

**Fig. 3:**
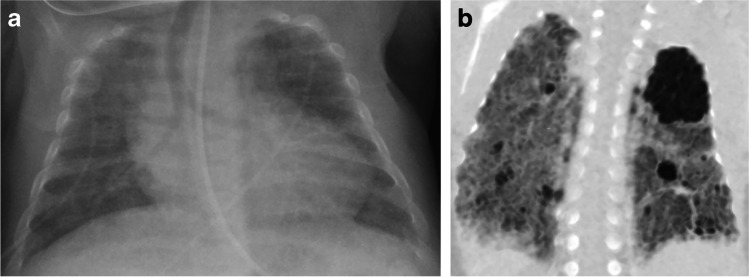
Lung findings of bronchopulmonary dysplasia on radiography and CT. **a** Anteroposterior (AP) chest radiograph of a premature infant girl born at 23 weeks of gestation, now post-menstrual age 38 weeks (15 weeks old). Portable chest radiograph shows diffuse increased density throughout the lungs but no evidence of air-trapping or cystic changes. **b** Coronal oblique minimum-intensity projection reconstructed CT image demonstrates hyperlucent lung in the left upper lobe and small cysts throughout the lungs. CT also suggested tracheomalacia was present (not shown)

The role of chest CT in preterm infants during their period of neonatal intensive care is largely based in problem-solving for more complex cases (and the same is true later in life), but numerous studies have evaluated the lung parenchyma and related the findings to severity of BPD or outcomes. These studies are split between early life and later life. The lung findings of BPD on CT are a recapitulation of those on chest radiograph, but with greater detail and richness (Fig. 3). Several studies laid the groundwork for CT scoring [34–38]. We focus here on the more recent CT studies because they reflect the current post-surfactant era of care.

The common thread among all of these CT studies was the objective findings on either high-resolution CT or chest CT. Researchers brought these all together in a relatively simple scoring system that related the score to the severity of BPD and, to a lesser degree, outcome [39, 40]. Hyperaeration of the lung, including global and focal air-trapping and mosaic perfusion, has been a common theme of all of these studies [34–40] and continues to be a primary theme [41–51]. A second category is “emphysema,” which Ochiai et al. [40] described as a sharply demarcated area of low attenuation. However, later-in-life reports of “emphysema” are variable as to the presence of this finding and description [37, 38]. There is also speculation that the emphysema identified in older children and young adults might be progressive [47, 48], but there is not proof of this. The third category is structural change, which includes linear opacities, triangular subpleural opacities and atelectasis/consolidative opacities [40, 47].

There is a substantial difference between the appearance of the lungs early in life [5, 40, 41, 49] when the infants first become stable enough for imaging, and later in life when the lungs have had time to heal and further develop. In general, the size and number of pulmonary opacities decrease as the child matures [49]. Simpson et al. [48] reported bronchial wall thickening and exposure to tobacco smoke as the most significant predictors of pulmonary functional decline in school-age children with a history of preterm birth, hypothesizing that the thickening reflected more inflammation or bronchial reactivity.

Dynamic-volume CT or cine CT is a method of using CT to look at the lung over a short period of time using a very low dose for each rotation of the CT to collect imaging data. The advantage is that this type of CT can show changes in the lungs and large airway over a respiratory cycle [52, 53]. This has been described as a method for choosing positive end-expiratory pressure in children with BPD and tracheobronchomalacia [52], which is a critical component related to preterm morbidity [53] and can also be used to evaluate regional lung ventilation [54].

The use of CT early in BPD is most limited by transportation of unstable infants to the CT scanner and to a lesser extent by concern about radiation. However, the association of CT findings to clinical course, pulmonary function and disease progression suggests that CT could play an increasing role in evaluating preterm babies that goes beyond problem-solving. A non-contrast chest CT performed at discharge from the NICU with a non-sedated feed-and-swaddle technique has the potential to provide information that would identify suspected findings, predict the early clinical course, and potentially decrease hospitalization in the first year after birth [8, 55].

## Chest magnetic resonance imaging

In recent years, chest MRI in preterm infants has been implemented to provide clinically meaningful information using readily available sequences, such as a fast gradient echo (GRE) and spin echo, that can evaluate lung tissue in infants with lung disease without requiring ionizing radiation [56, 57]. Walkup et al. [56] showed that GRE images demonstrate a significantly greater volume of high-intensity lung (putatively combinations of fibrosis, edema, consolidation and atelectasis) in infants with BPD compared with full-term infants and preterm infants who were not diagnosed with BPD. However, the volume of low-intensity lung did not discriminate clinical BPD diagnoses, despite the established presence of low-density lung pathologies in BPD such as alveolar simplification, cysts, emphysema and air-trapping. This is likely attributable to that fact that conventional Cartesian sequences typically cannot achieve minimum echo times (TE) below the lung parenchymal T2* (~ 0.5–3 ms at typical field strengths) and thus do not provide sufficient image contrast between short-T2* tissues and true cystic/air-trapped regions [58]. Even so, radiologic reader scores from a modified Ochiai system that evaluates parenchymal abnormalities (discussed in detail later) [40] significantly delineated all three groups of preterm infants with BPD, preterm infants without BPD, and term infants.

**Fig. 4:**
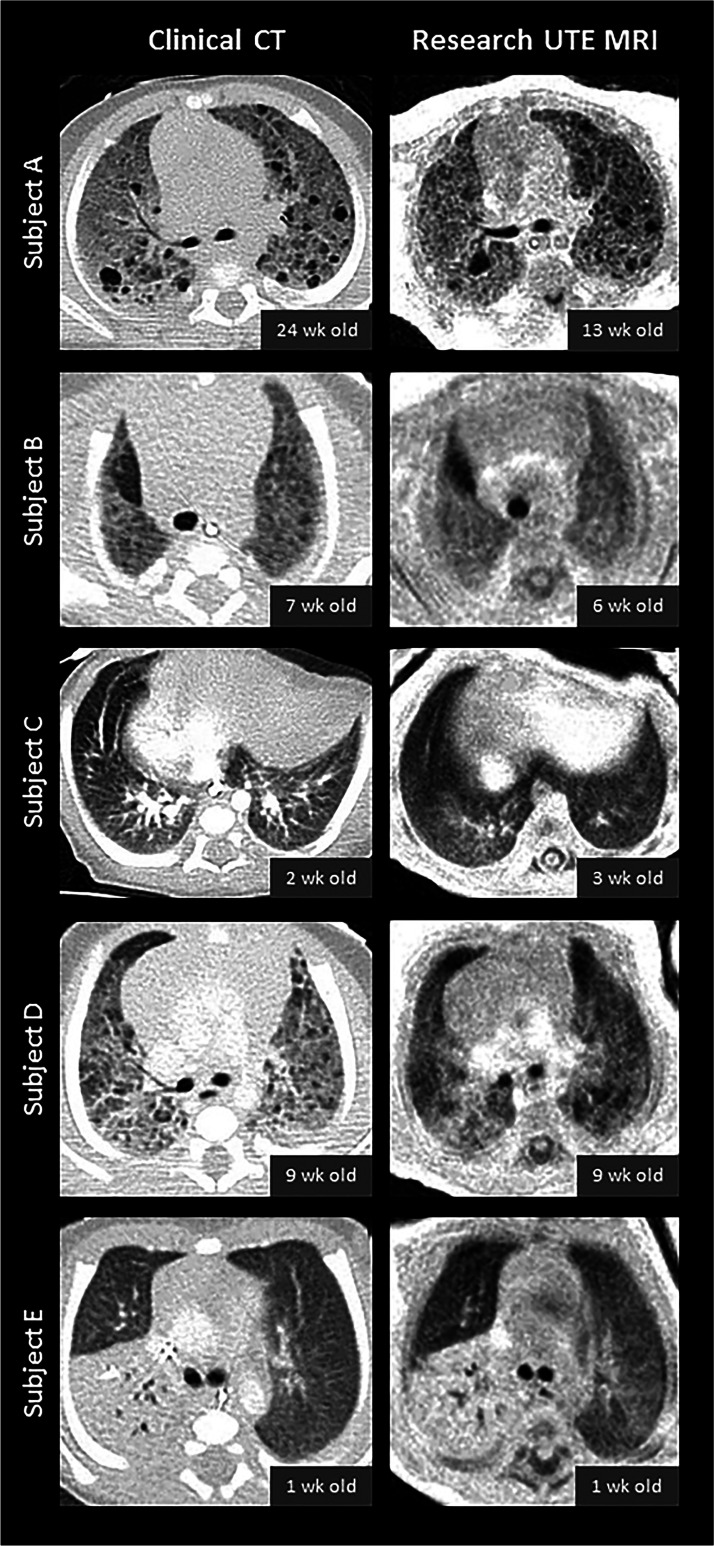
Comparison of five neonates’ matched axial CT and axial ultrafast echo-time (UTE) MRI slices obtained at a similar time visually demonstrates the similarity of tissue density and lung findings. Subject A is a neonate with severe bronchopulmonary dysplasia (BPD). The quantitative density of UTE is highly correlated with CT density in the lung. Note the different appearance in the spinal canal on the MRI caused by the different T1 and T2^*^ properties in the spinal canal compared to the lung tissue. Reproduced with permission [64]

**Fig. 5:**
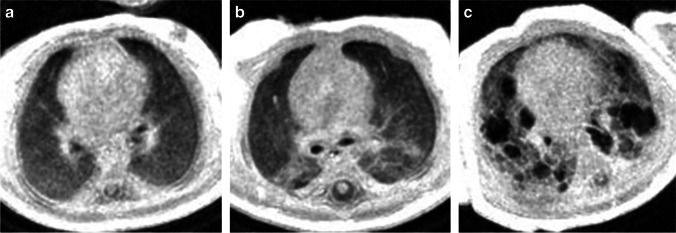
Axial ultrashort echo-time (UTE) MRI and modified Ochiai score. **a–c** Examples of preterm female born at 26 weeks of gestation, now post-menstrual age 38 weeks (12 weeks old) with mild (**a**), preterm female born at 25 weeks of gestation, now post-menstrual age 41 weeks (16 weeks old) with moderate (**b**) and preterm male born at 23 weeks of gestation, now post-menstrual age 40 weeks (17 weeks old) with severe (**c**) bronchopulmonary dysplasia on axial UTE MR images of the lung based on the modified Ochiai score. The mild patient (**a**) was scored as 1 out of 14, the moderate patient (**b**) 6 out of 14 and the severe patient (**c**) 12 out of 14. Increasing score was positively correlated with greater need for respiratory support and short-term clinical outcomes

Recent technical developments in sequence acquisition have opened the door to pulmonary MRI using center-out 3-D k-space acquisitions with ultrashort echo times (UTE) and zero echo times (ZTE) [9, 59–63]. Unlike Cartesian sequences, these center-out sequences are inherently robust to subject motion, reducing the need for sedation/anesthesia. Further, these short-TE acquisitions have strong proton-density weighting, allowing for more accurate visualization and quantification of both hyper- and hypodense lung parenchymal tissue. Nozawa et al. [64] demonstrated the potential for ZTE MRI (pointwise encoding time reduction with radial acquisition, or PETRA) to acquire CT-like proton-density-weighted pulmonary images in two infants with congenital cystic lung disease, which is likely to be similarly effective when implemented in infants with lung disease of prematurity. Further, our Center for Pulmonary Imaging Research at Cincinnati Children’s Hospital has demonstrated that regional lung intensity on UTE MRI is quantitatively comparable to lung density on chest CT (Fig. 4) in individual neonates with lung disease who underwent both MRI and CT [65]. Using this UTE MRI method, Higano et al. [65] examined whole-lung density distributions in 38 infants with and without BPD and found that the percentage of lung volume with abnormally hyperdense and hypodense tissue density (defined as >+1σ or < –1σ, respectively, compared to an average control distribution) correlated moderately with clinical BPD severity diagnosis (R^2^ = 0.21) but more strongly with respiratory support at discharge from the NICU (R^2^ = 0.45). In an additional study, Higano et al. [66] showed that modified Ochiai scoring of paired UTE and GRE lung images from neonates with BPD (Fig. 5) correlated with duration of various respiratory support levels more strongly than any individual clinical measure (ventilator support, R^2^ = 0.86; positive pressure ventilation, R^2^ = 0.91; any support, R^2^ = 0.77).

**Fig. 6:**
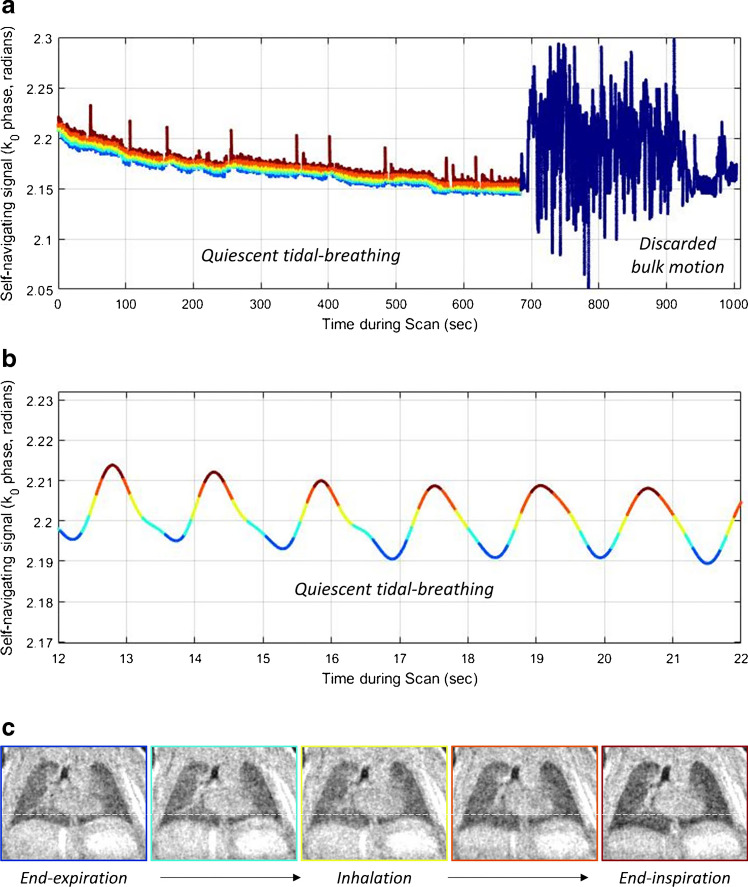
Preterm female born at 27 weeks of gestation, now post-menstrual age 36 weeks (9 weeks old). Self-navigating signal obtained from the center of ultrashort echo-time (UTE) MRI k-space (k_0_). **a** Graph illustrates the difference in the signal between the signal obtained in quiescent tidal breathing (*multicolored line*) and when the child is moving significantly (*dark blue*). This allows data from bulk motion to be discarded. **b** Graph is a magnification of a smoothed and binned signal of the quiescent tidal-breathing signal. The colors of the segments in the line correspond to the phases of respiration. Blue is end-expiration and dark maroon is end-inspiration. **c** Each respiratory bin can then be reconstructed into an image during a different phase of respiration, which is shown in the line of coronal UTE MRI lung images. The movement of the diaphram and chest wall, and the change in lung density, is illustrated. This has been shown to have potential for regional ventilation maps

As MRI strategies emerge to evaluate neonatal lung structure, so, too, do complementary MRI strategies for measuring lung function. UTE MRI sequences inherently acquire the center of k-space (k_0_) at every acquisition, which can be exploited as a self-navigating signal for respiratory gating (Fig. 6) that allows raw data to be binned and selectively reconstructed at different phases of the respiratory cycle [67–71], often from a child who was scanned during tidal breathing. Advanced reconstruction strategies, such as iMoCo (iterative motion-compensation reconstruction) UTE MRI, have demonstrated high-scanning efficiency, sharper anatomical lung structures and higher apparent signal-to-noise ratio compared to other motion-correction methods in free-breathing pulmonary MRI of adults and pediatrics. Zhu et al. [72] showed the feasibility of neonatal iMoCo UTE in one non-sedated 10‐week‐old with pulmonary interstitial glycogenosis, demonstrating the ability to capture and reject non-compliant bulk motion. Using dynamic images throughout the breathing cycle, various respiratory metrics of interest have been measured in neonates with lung disease, including tidal volume, hyperexpansion, global minute ventilation, degree of central airway collapse and tracheal work of breathing [69, 73–76]. By measuring local changes in signal intensities or anatomical deformation throughout the breathing cycle, dynamic pulmonary MRI strategies also open the door to quantitative lung ventilation, similar to techniques used with chest CT [77], but during free-breathing and without ionizing radiation. Capaldi et al. [78] recently implemented convolutional neural networks in deep learning to generate synthetic ventilation images from free-breathing pulmonary MRI in adults with various lung diseases, with a correlation between ventilation defect percentage from MRI and forced expiratory volume in 1 s (FEV_1_) from spirometry (*P* < 0.001); similar strategies might be readily translatable to evaluating pulmonary function in the neonatal population.

While tidal-breathing ^1^H chest MRI strategies are emerging to indirectly measure regional ventilation, hyperpolarized inhaled noble-gas (^3^He and ^129^Xe) MRI has been used for several years as a more direct ventilation metric, with established safety [79–81] and numerous applications in a variety of adult and pediatric lung disorders [82–84]. Altes et al. [85] recently developed a gas ventilation imaging protocol for MRI acquisition sequences and gas-delivery strategies in a proof-of-concept study with seven non-sedated children younger than 4 years, including one healthy infant, three infants with cystic fibrosis, and one infant born prematurely at 28 weeks of gestation; in this small feasibility study, focal ventilation defects were clearly identified in children with respiratory disease. Hyperpolarized gas MRI can also probe the length-scales of microstructural alveolar airspaces by detecting Brownian airspace diffusion of inhaled gas particles [86]. This technique has been implemented in older pediatric survivors of preterm birth [87] and in an explanted neonatal lung specimen with filamin-A mutation (closely representing BPD lung disease) [88], with significantly elevated apparent diffusion coefficient (ADC) values in children with disease compared to age-matched controls demonstrating enlarged gas-exchange units. While not yet implemented in live neonates, inhaled gas diffusion MRI has strong potential to elucidate abnormal and interrupted alveolar development in infants born preterm.

## Trachea and large airways

In addition to the arrest in development of the pulmonary parenchyma, neonates who are born prematurely have disruption in development of the central airway. The net impact of preterm birth is that the central airway is smaller, more compliant and more susceptible to airway injury, especially in neonates who require endotracheal intubation and positive pressure ventilation [89–92]. Consequently, disease of the central airway is quite common in neonates with BPD. Central airway disease can be divided into dynamic and fixed central airway pathology and results in increased respiratory morbidity in these fragile infants.

**Fig. 7:**
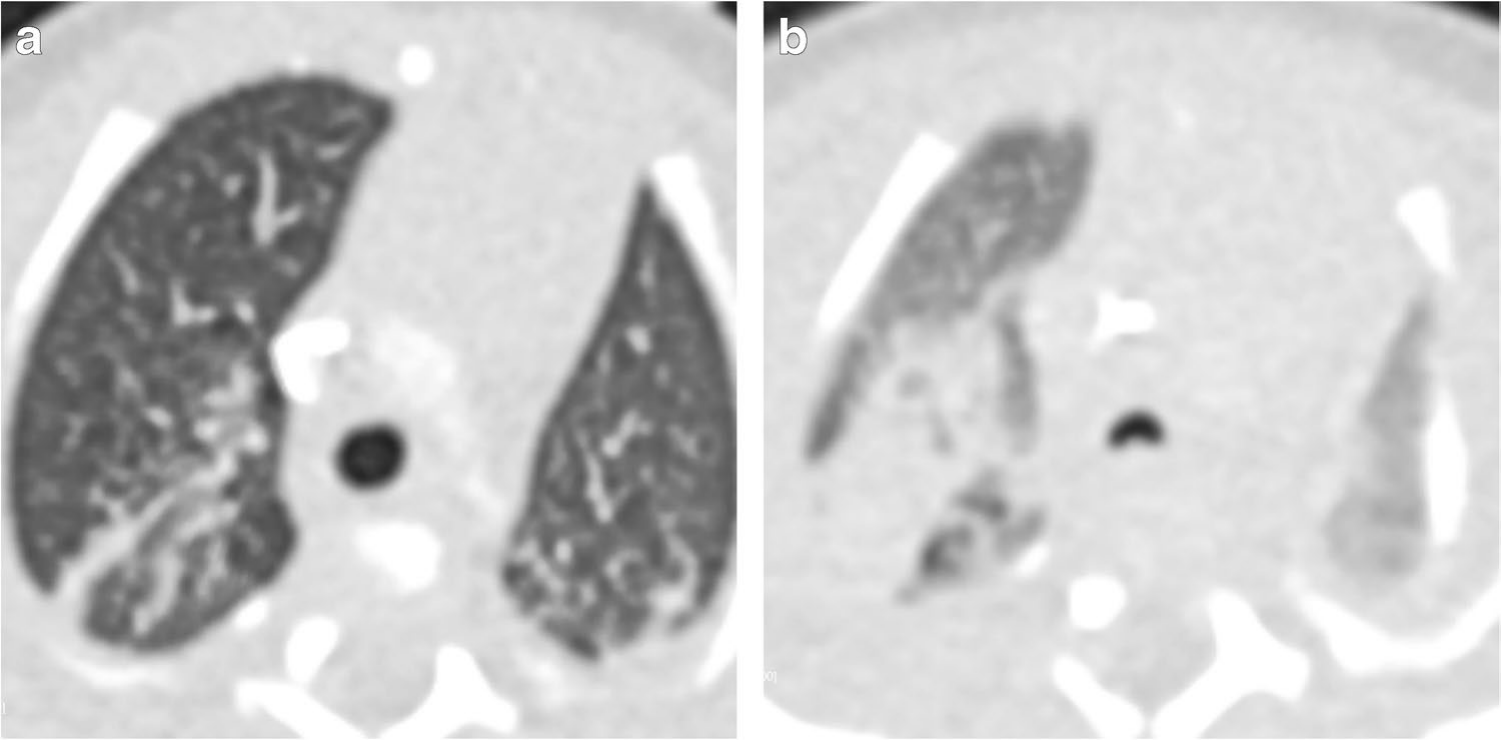
Airway pressure imaging in a preterm boy born at 25 weeks of gestation, now post-menstrual age 31 weeks (6 weeks old). **a, b** Axial CT demonstrates the difference in the appearance of the lungs and trachea when imaged with 14 cm of water-positive end-expiratory pressure (PEEP) (**a**) and without PEEP (**b**). Airway pressure support is often needed but can mask tracheobronchomalacia when it is present. Removing the pressure support at the time of imaging can be very helpful in evaluating the lungs and the airway when performing CT

Dynamic airway lesions such as tracheobronchomalacia are identified in 10–48% of neonates with BPD undergoing bronchoscopy, which is the reference standard for diagnosis [53, 75, 93, 94]. However, the true prevalence is unclear because children undergoing bronchoscopy are a heavily biased subset, and many neonates who do not undergo bronchoscopy have dynamic airway collapse (Fig. 7) based on advanced imaging techniques [74]. Tracheobronchomalacia is associated with increased morbidity during the initial hospital admission with prolonged need for mechanical ventilation, longer hospitalization and increased need for tracheotomy [53]. Following discharge, children with tracheobronchomalacia require more medical therapies and feeding support [53] and have increased frequency of rehospitalization, particularly during respiratory illnesses [95].

**Fig. 8:**
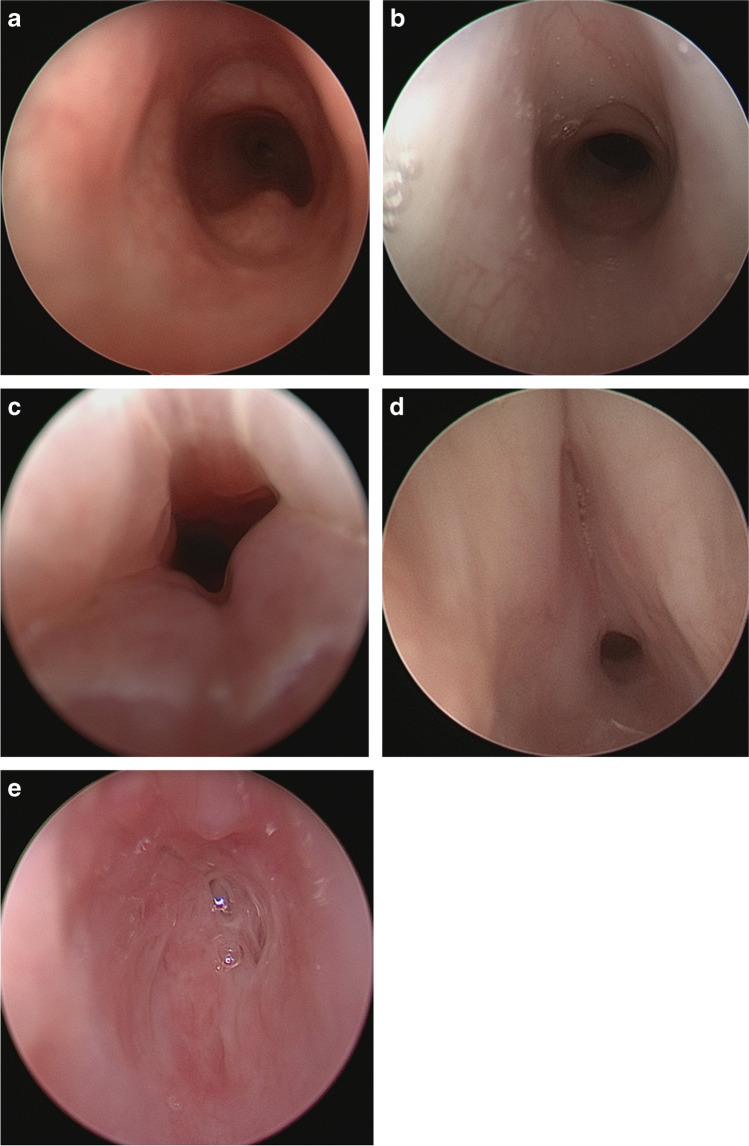
The Myer-Cotton scale. **a–e** Endoscopic images of infants with bronchopulmonary dysplasia (BPD) demonstrate a normal subglottis (**a**) followed by examples of the Myer-Cotton subglottic stenosis grading system. Grade 1 is defined as < 50% obstruction (**b**), grade 2 as 51–70% obstruction (**c**), grade 3 as 71–99% obstruction (**d**) and grade 4 as complete 100% obstruction (**e**)

Fixed airway lesions such as subglottic stenosis are also common in those neonates who require invasive mechanical ventilation. The cricoid is the narrowest portion of the airway and is particularly susceptible to injury from intubation in neonates. The prevalence of post-intubation subglottic stenosis in neonates is 0.9–8.3% [96] and is likely higher in those with BPD than the general neonatal population because of the small airway size, need for prolonged intubation, and multiple intubation attempts [97]. As with tracheobronchomalacia subglottic stenosis is diagnosed by bronchoscopy and utilizes the Myer-Cotton scale (Fig. 8), which grades severity based on the largest endotracheal tube that permits an air leak at 20 cm H_2_O [98]. Depending on the severity of subglottic stenosis, treatment options range from observation or endoscopic balloon dilation for milder stenosis to open airway surgery or tracheotomy for more severe disease [99, 100].

Despite the impact of central airway disease on children with BPD, our understanding is limited because the diagnosis typically relies on direct visualization with bronchoscopy. However, recent advances in imaging technologies such as CT and MR can provide a noninvasive way to assess the neonatal airway without sedation. In the case of MR, this can be done without ionizing radiation and thus might be suitable for longitudinal population studies of central airway disease in BPD.

## Central airway magnetic resonance imaging

**Fig. 9:**
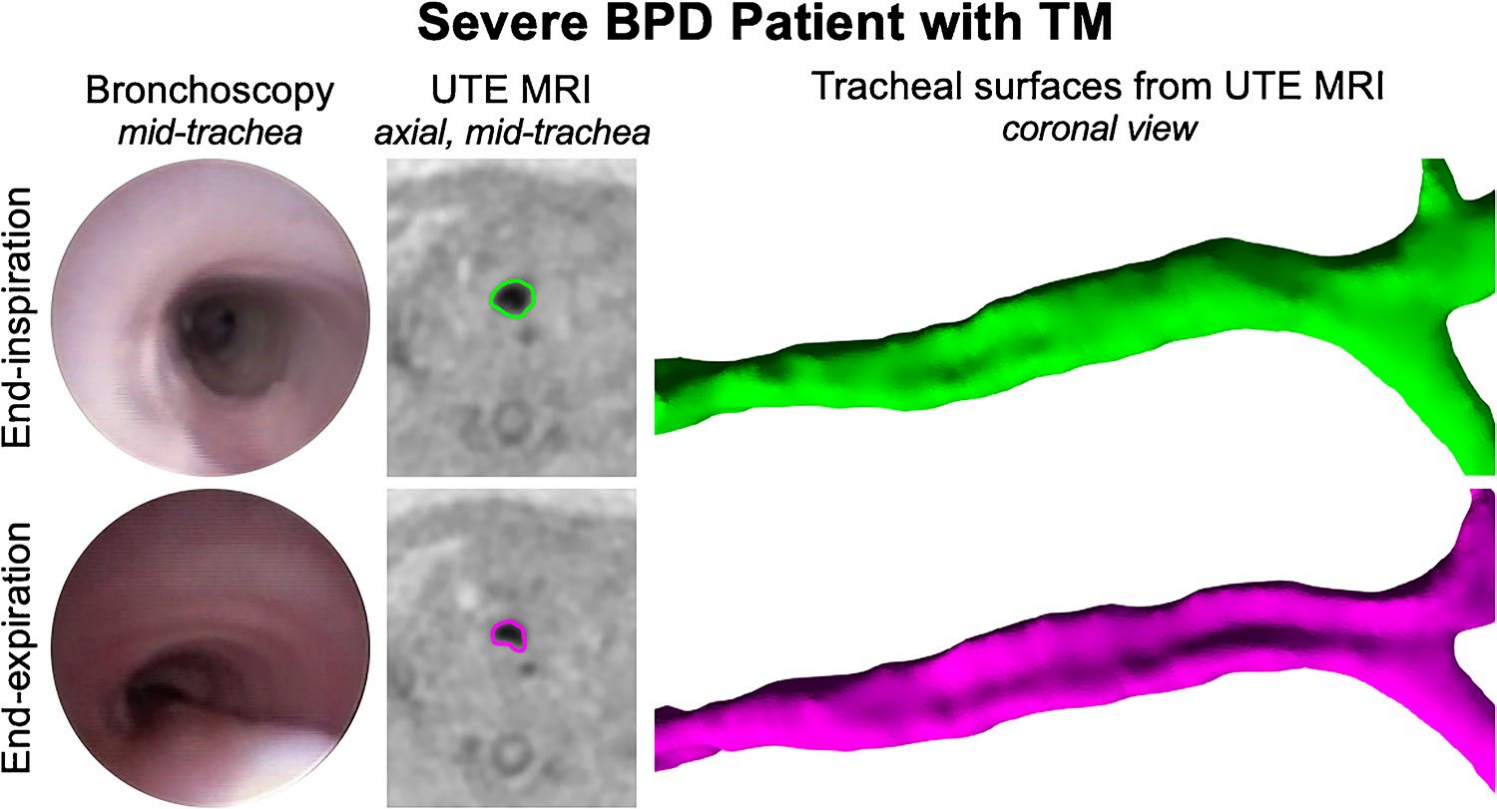
Assessing the cross-sectional area of the tracheal lumen based on ultrashort echo-time (UTE) MRI in a preterm girl born at 25 weeks of gestation, now post-menstrual age 38 weeks (13 weeks old) with severe bronchopulmonary dysplasia and tracheomalacia. Bronchoscopic axial UTE MR images (*left*) and three-dimensional (3-D) surface-rendered images (*right*) show a relatively round trachea at end-inspiration (*top row*) and inward bowing of the posterior trachealis membrane on end-expiration (*bottom row*)

Magnetic resonance imaging has been used to noninvasively investigate airway abnormalities such as tracheobronchomalacia and subglottic stenosis in neonates and animal models of neonates [101–103]. Retrospective respiratory gating of UTE MRI, the same sequence that evaluates the lung parenchyma, simultaneously allows measurement of 3-D dynamic changes in lumen size [74]. Assessing the cross-sectional area of the tracheal lumen based on UTE MRI (Fig. 9) has been shown to diagnose tracheomalacia with equivalent accuracy to bronchoscopy [75].

**Fig. 10:**
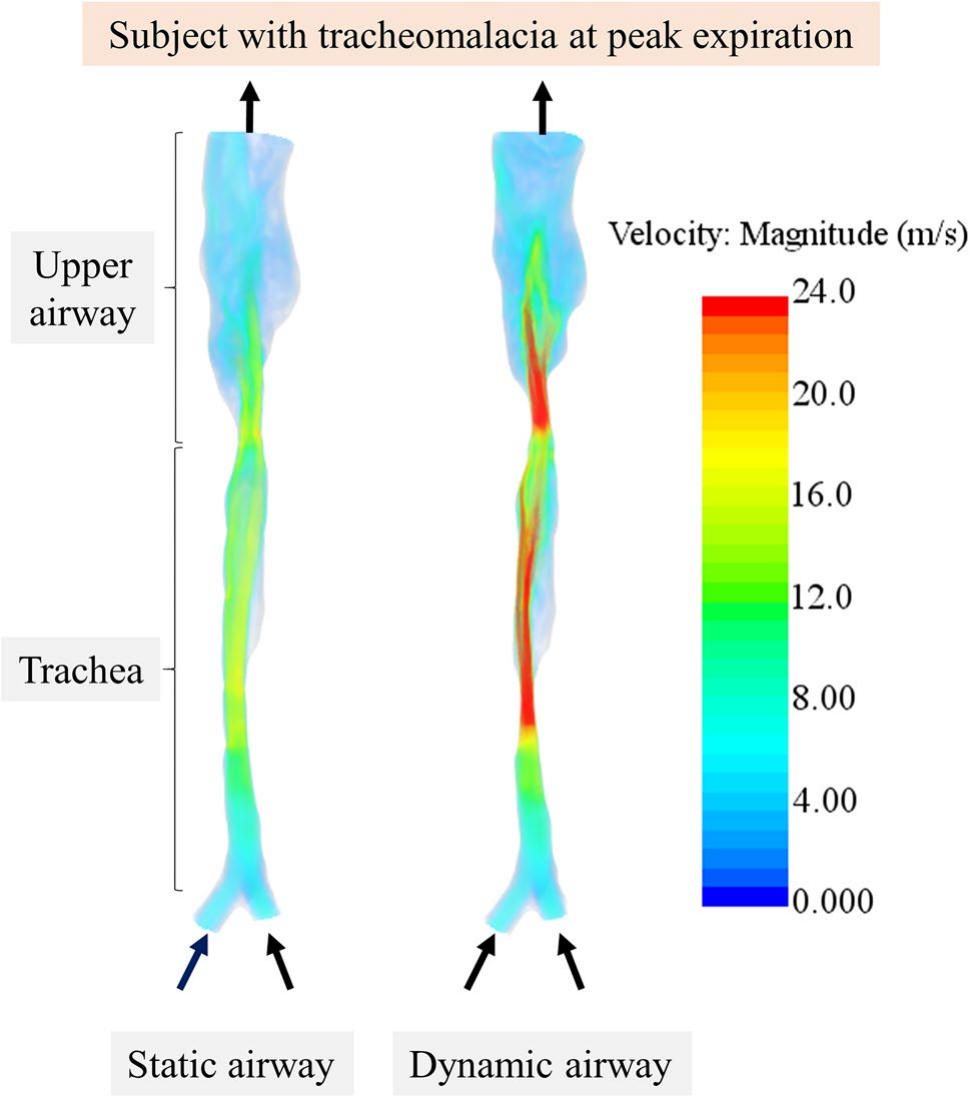
Using MRI-derived images and airflow volume, the trachea can be modeled as a non-dynamic structure (*static airway, left*) and a dynamic structure (*dynamic airway, middle*). The dynamic structure modeling results in a high-velocity jet formed in the middle of the trachea caused by narrowing during expiration, which increases work of breathing. The static airway represents the largest airway for that subject observed during breathing and represents breathing without tracheomalacia, which provides the least work of breathing. Arrows indicate the airflow direction from the main bronchi to nasopharynx. Velocity key is on the right

Until recently, MRI has only been used to depict anatomy and the significance of the tracheomalacia has been inferred, but tracheomalacia likely has a profound effect on energy needs of these tiny preterm babies. Using MRI as a technique for imaging airway anatomy and motion, computational fluid dynamics can calculate airflow parameters such as airway resistance or pressure gradients based on airway anatomy, motion and MRI-derived tidal volumes (Fig. 10). Using this approach, Gunatilaka et al. [76] demonstrated that neonates with tracheomalacia used more than 10 times the energy to breathe that they would have with static airways.

A similar approach of imaging based computational fluid dynamics has also been used to assess the effect of subglottic stenosis on neonatal breathing, revealing that the increased airway resistance caused by the stenosis is associated not only with the degree of stenotic narrowing [104, 105] but also with the axial position of the stenosis [75].

## Central airway computed tomography

As a more readily available modality, CT is much more commonly used to assess for tracheobronchomalacia and other central airway anatomies in infants with BPD, using a relatively low radiation dose, with airway abnormalities detected from scans exposing subjects to less than 2 mSv [106].

Various studies have used CT to diagnose tracheobronchomalacia in infants with and without BPD with sensitivity values ranging 75–100% (and as low as 37.5% for tracheomalacia alone) and specificity of 48–97% [107–109]. Differences in these results stem from differences in the age range of children, the use of static or dynamic CT, the phase of breathing or breathing maneuver in which imaging was acquired, and the technique used to determine the presence of tracheobronchomalacia from CT images. The change in tracheal lumen cross-sectional area at the point of greatest collapse is higher in infants with tracheobronchomalacia than in those without tracheobronchomalacia [55, 110, 111]. However, another study found CT is less sensitive than tracheobronchography in the diagnosis of tracheomalacia in ventilator-dependent infants and exposes them to a higher radiation dose [112]. In addition to the detection of tracheobronchomalacia, dynamic CT provides enough fidelity in airway measurement to be used to set ventilator-positive end-expiratory pressure settings [52].

## Pulmonary vasculature and pulmonary hypertension

Bronchopulmonary dysplasia is associated with pulmonary vascular disease and pulmonary hypertension because of alterations in development of pulmonary parenchyma and vasculature [113–115]. The presence of cor pulmonale was noted by Northway et al. [10] in the autopsy specimens of five long-term survivors of preterm birth. Currently, there is a renewed interest in BPD-associated pulmonary hypertension. The incidence of BPD–pulmonary hypertension increases with severity of BPD, with 20–40% incidence of pulmonary hypertension in infants with severe BPD [95, 116]. Infants with BPD-associated pulmonary vascular disease and pulmonary hypertension have worse outcomes, including longer duration of respiratory support [95, 114, 117], increased incidence of respiratory disease in early childhood [113] and increased mortality [94, 114, 117–120] compared to infants with BPD but without pulmonary vascular disease or pulmonary hypertension. The increased morbidity and mortality associated with BPD pulmonary vascular disease and pulmonary hypertension has resulted in research focused on the early identification and diagnosis, risk stratification and pharmacological therapy for these children [3].

While cardiac catheterization remains the gold standard for diagnosing pulmonary hypertension [121], echocardiography is the predominant modality for screening and monitoring BPD–pulmonary hypertension. Echocardiographic evidence of pulmonary vascular disease at 7 days of age was associated with respiratory disease in early childhood [114], and current guidelines recommend screening in all infants at the time of BPD diagnosis [122]. Echocardiography evaluation of BPD-associated pulmonary hypertension should include a complete anatomical survey; assessment of right and left ventricular size, function and hypertrophy; right ventricular pressure; and presence of any anatomical shunts [122]. In particular, the presence and size of a patent ductus arteriosus is of particular importance. Evaluation for BPD-associated pulmonary hypertension should focus on quantitative indices of right ventricular pressure including tricuspid regurgitant jet velocity, shunt gradient and left ventricular eccentricity index and right ventricular function, including tricuspid annular plane systolic excursion, right ventricular fractional area change, and right ventricular longitudinal strain [123]. Comprehensive echocardiographic evaluation should also include screening and evaluation of pulmonary venous return, especially presence of stenosis. Isolated or multi-vessel stenosis might be more common in the premature infant and is an independent predictor for, and an additional pathological driver of, the development of pulmonary vascular disease. It is critically important that these echocardiographic parameters be assessed in full during each echocardiographic study if possible [124, 125]. Nevertheless, assessment of these anatomical and physiological markers is often limited secondary to lung hyperinflation, cardiac malposition and poor imaging windows. Often, providers rely on qualitative markers of right ventricular hypertension (including interventricular septal flattening, which is sensitive but not specific for pulmonary hypertension and subject to interobserver variability) and qualitative assessment of right ventricular function.

Because of these limitations, additional diagnostic studies including lung perfusion scan, cardiac catheterization, chest CT or cardiac MRI should be considered when diagnostic uncertainty persists. Lung perfusion scan provides for assessment of maldistribution of pulmonary blood flow, which can result in increased pulmonary vascular resistance, and is an adjunctive diagnostic study for evaluating pulmonary vein stenosis [126]. Cardiac catheterization should be performed whenever there is concern regarding the degree of intracardiac shunting, branch pulmonary artery and pulmonary vein anatomy, left ventricular diastolic dysfunction, presence of aortopulmonary collaterals, and certainly prior to escalation of pulmonary vasodilator therapy [121]. Acute vasodilator testing is also performed during cardiac catheterization and might help guide therapy [127].

**Fig. 11:**
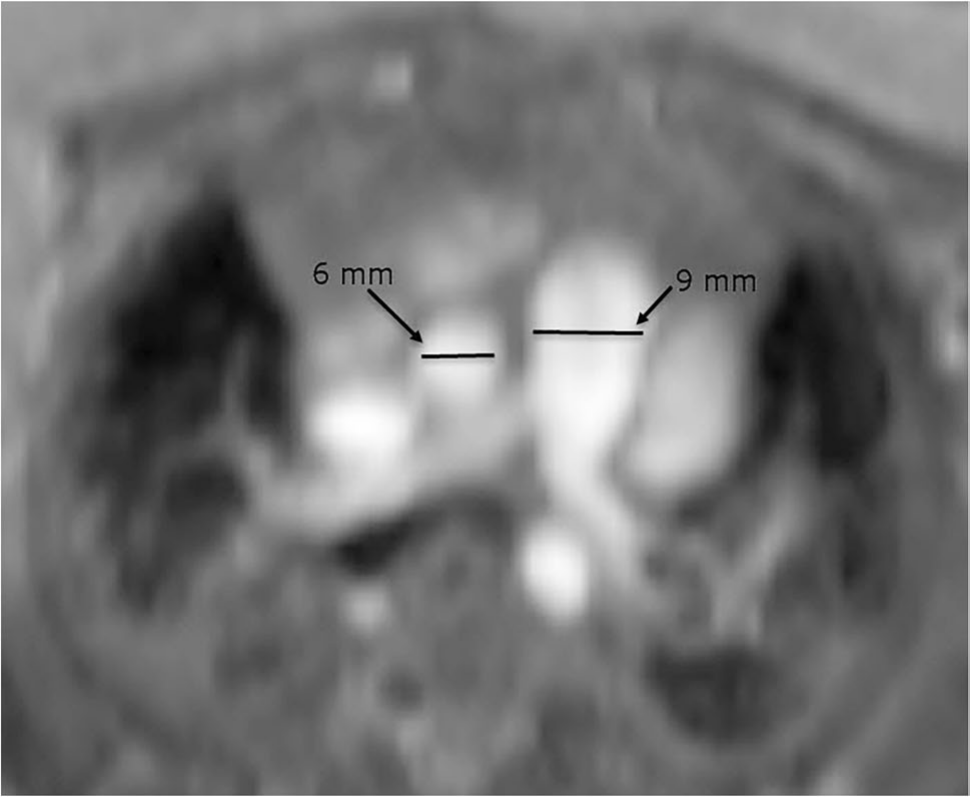
Morphological markers of pulmonary hypertension in a girl born at 26 weeks of gestation with MRI performed at post-menstrual age 38 weeks (12 weeks old). Axial MRI bright-blood image through the main pulmonary artery and the ascending aorta demonstrates a 9-mm main pulmonary artery and a 6-mm aorta, resulting in a ratio of 1.5:1. A ratio of 1.3:1 or greater, in the absence of a larger left-to-right shunt, is very specific for pulmonary hypertension

Imaging with chest CT is used in both adult and pediatric populations to assess for evidence of vascular malformations, pulmonary veno-occlusive disease, interstitial lung disease and chronic thromboembolic disease at the time of pulmonary hypertension diagnosis [121]. Additionally, chest CT has been used to assess morphological markers of pulmonary hypertension including the main pulmonary artery size and main pulmonary artery to aorta (PA/AO) ratio (Fig. 11) [128–130]. No studies have investigated the role of chest CT in the diagnosis of BPD-associated pulmonary hypertension. However, del Cerro and colleagues [131] found that 19/29 (66%) infants with BPD-associated pulmonary hypertension had aortopulmonary collaterals, pulmonary vein stenosis, atrial septal defect or patent ductus arteriosus diagnosed with CT or catheterization. Additionally, CT was shown to aid in the diagnosis of pulmonary vein stenosis in infants with BPD in a multicenter retrospective study [124]. These data suggest that CT could have a clinical role in identifying vascular pathology or characterizing shunt lesions prior to surgical or percutaneous intervention in the BPD-associated pulmonary hypertension population.

Cardiac MRI allows for evaluation of cardiac morphology, ventricular size and function, cardiac output, and pulmonary blood flow distribution. Assessment with cardiac MRI is used in the adult and pediatric pulmonary hypertension populations where MRI-derived right ventricular function has been associated with mortality [132, 133]. In a cohort of 52 infants, the PA/AO ratio was associated with BPD severity, duration of respiratory support, hospitalization length and need for pulmonary vasodilator therapy [134]. In that study, ventricular size, ventricular function, cardiac output and pulmonary blood flow could be determined but were not associated with BPD severity or short-term clinical outcomes.

**Fig. 12:**
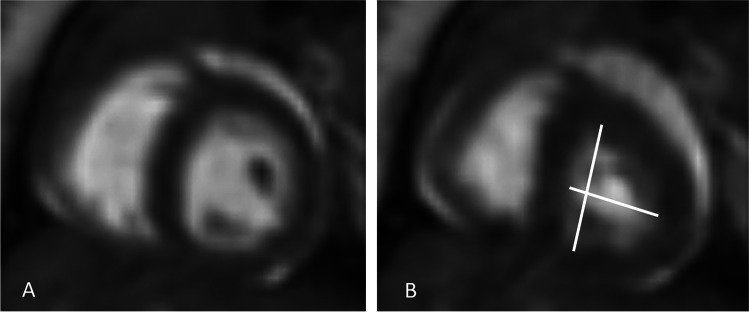
Left ventricular eccentricity index in a boy born at 24 weeks of gestation and imaged at post-menstrual age 40 weeks (16 weeks old). **a, b** MRI short-axis cine steady-state free precession (SSFP) slice at the level of the papillary muscles shows diastole (**a**) and systole (**b**). During systole, the interventricular septum is clearly flattened, creating a D-shape left ventricle. A left ventricular eccentricity index greater than 1.3:1 is diagnostic of pulmonary hypertension

**Fig. 13:**
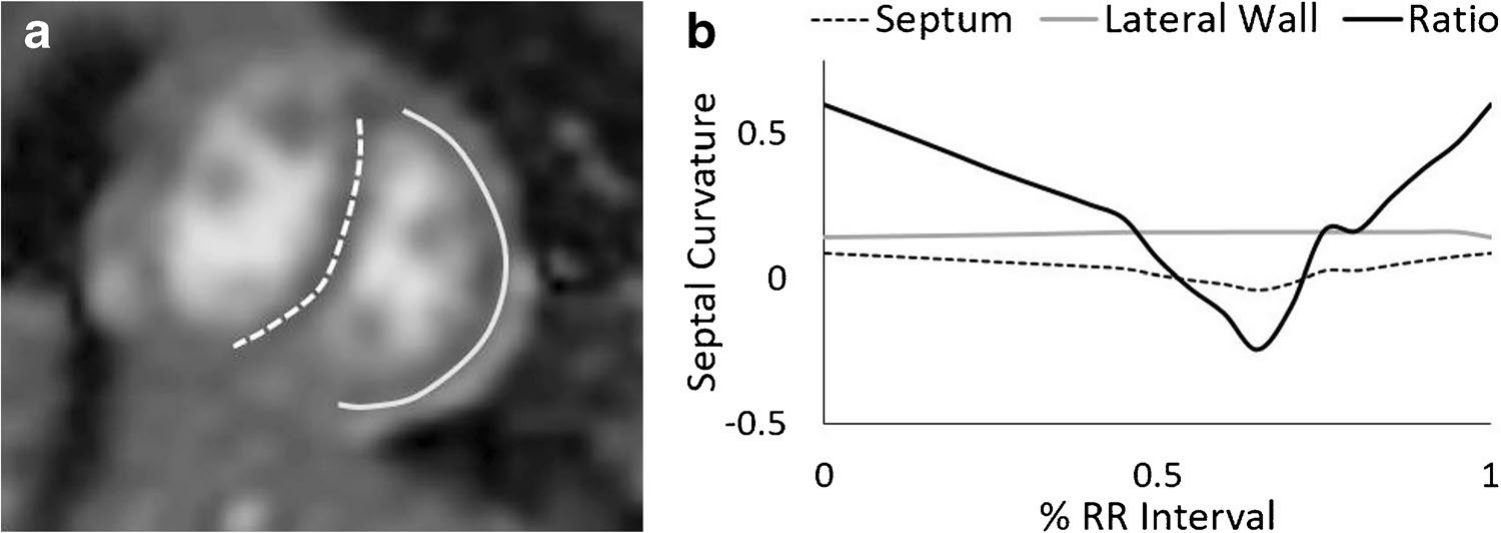
Preterm male born at 24 weeks of gestation, now post-menstrual age 31 weeks (7 weeks old). **a**, **b** MR septal curvature is derived as the ratio of curvature of the interventricular septum (*dashed line*) and lateral wall (*solid line*) throughout the cardiac cycle (**a**). MRI short-axis cine steady-state free precession (SSFP) image shown here demonstrates the phase in the cardiac cycle at which minimum septal curvature occurred (**a**). In this case the interventricular septum is everted, resulting in a negative septal curvature value. Septal curvature throughout the cardiac cycle for the interventricular septum (*dashed line*), lateral wall (*gray line*), and ratio of the interventricular septum to the lateral wall (*black line*, **b**)

Cardiac MRI markers of right ventricular afterload have been associated with invasive hemodynamics in the pediatric pulmonary hypertension population [135, 136]. Left ventricular eccentricity index (Fig. 12), a quantitative marker of interventricular septal flattening that is typically measured at end-systole, was associated with duration of respiratory support, hospitalization length and need for pulmonary vasodilator therapy in infants with BPD [134, 137]. Further, interventricular septal curvature (Fig. 13), which provides a quantitative evaluation of septal flattening throughout the cardiac cycle, demonstrated improved discrimination of need for pulmonary vasodilator therapy compared to other clinical and cardiac MRI indices [137].

Management of BPD-associated pulmonary hypertension focuses on providing adequate respiratory support; treatment of hypoxia, infection, aspiration and airway disease; followed by treatment with pulmonary vasodilator therapy if pulmonary hypertension persists [121]. Cohen et al. [138] reported on a cohort of 269 pediatric patients treated with sildenafil, including 135 infants with BPD-associated pulmonary hypertension. Mortality in recent BPD-associated pulmonary hypertension cohorts was improved compared to historical cohorts [121, 138, 139]. Additionally, 45% of these children were able to discontinue sildenafil therapy after improvement in pulmonary hypertension. While these results are encouraging, infants with BPD-associated pulmonary hypertension remain at risk for developing pulmonary vascular disease and pulmonary hypertension later in life. Recently preterm birth, in the absence of BPD, has been associated with pulmonary vascular disease in asymptomatic children and young adults [140, 141] supporting lifelong monitoring for pulmonary vascular disease and pulmonary hypertension in this at-risk population.

## Summary — putting it all together

Chronic respiratory disease of prematurity is best thought of as a disease that affects all components of the cardiopulmonary system: the lung parenchyma, small airways, large airways, pulmonary vasculature and heart. This creates the ability to “phenotype” infants based on the disease presence and severity of each component [55]. Among children with severe BPD, tracheobronchomalacia and pulmonary hypertension are also likely to be present, but a child with mild lung disease could also have tracheobronchomalacia or pulmonary hypertension. Such a child could be predicted to have a more difficult course after discharge than a preterm baby without an additional comorbidity. Therefore, knowledge of the child’s phenotype from imaging is very important.

Chest radiograph is very limited in its ability to characterize all three aspects of disease — BPD, tracheobronchomalacia and pulmonary hypertension — but a bubbly cystic appearance with coarse opacities intermixed with hyperlucent areas does predict severity and is now part of the BPD definition [3]. However, it does not provide much information about the airway or cardiovascular involvement that can have such an important impact on outcomes.

On the other hand, chest CT can provide detailed evaluation of the lungs for air-trapping, cysts, mosaic perfusion and opacities, which can range from linear bands and small triangular opacities against the pleura to large confluent opacities. Additionally, on CT the trachea is well evaluated for the presence or absence of tracheobronchomalacia. There are several methods to perform the chest CT: inspiration/expiration CT, inspiration only or during tidal breathing. Several papers in the review used inspiration/expiration techniques, but that usually requires sedation or general anesthesia [5]. Our recommendation based on experience is to image during tidal breathing using either feed-and-swaddle technique or minimal sedation if needed. Temporary removal of any sort of airway pressure support reveals the true nature of the trachea and bronchi.

The addition of contrast agent to a chest CT can be considered. Advantages are that the vessels can be visualized. The PA/AO ratio could indicate the presence of pulmonary hypertension, a ductus arteriosus or other left-to-right shunt, and could even help to evaluate interventricular septal flattening. The disadvantage of giving contrast agent is the potential for infant movement during injection. Additionally, CT angiography can be used to evaluate the pulmonary veins for stenosis. However, echocardiography remains the primary method of evaluating for pulmonary hypertension and pulmonary vein stenosis.

Magnetic resonance imaging of the lungs, heart and trachea is on the cusp of becoming a clinical reality, with UTE imaging playing a large role in providing CT-like imaging of the lung but also dynamic imaging of the trachea during tidal breathing. Imaging of the heart and vasculature can be performed without contrast agent. Thus, MRI can assess the PA/AO ratio, septal flattening and septal curvature, which is a very good measure of the severity of BPD–pulmonary hypertension [134, 137]. Evaluation of the pulmonary veins would likely require the use of contrast agent.

One of the exciting and novel uses of the dynamic UTE images of the lung is the ability to use the signal intensity over the respiratory cycle to determine regional ventilation [77]. A similar process could be done with dynamic CT data, but only over one or two breathing cycles. The advantage of the MRI is a much higher temporal resolution and a single composite breathing cycle created from several minutes of imaging and the absence of radiation [65].

Radiologists have always played a significant role in the care and treatment of preterm babies via a variety of imaging modalities. Imaging of the pulmonary system likely has played the greatest role for radiology and now there is new evidence that cross-sectional imaging has added value in characterizing the contributions of the lung, large airways and cardiovascular system to the child’s clinical status and in predicting morbidity.
